# Diet-induced obesity potentiates the growth of gastric cancer in mice

**DOI:** 10.3892/etm.2012.657

**Published:** 2012-08-07

**Authors:** HAI-JUN LI, XIANG-MING CHE, WEI ZHAO, SHI-CAI HE, ZHENG-LIANG ZHANG, RUI CHEN

**Affiliations:** 1 Department of General Surgery, First Affiliated Hospital of the Medical College of Xi’an Jiaotong University, Xi’an 710061;; 2 Department of Emergency, Second Affiliated Hospital of the Medical College of Xi’an Jiaotong University, Xi’an 710004, P.R. China

**Keywords:** gastric cancer, obesity, adipocytokine, visfatin, insulin resistance

## Abstract

Obesity increases the risk of gastric cancer and may affect its development and progression, however, the mechanisms underlying this association are completely unknown. The purpose of the current study was to investigate the effect of obesity on gastric cancer growth by adopting a novel *in vivo* model. Diet-induced obese and lean mice were inoculated with murine forestomach carcinoma cells, and studied for 2 weeks. Tumor histology, cellular proliferation and apoptosis were evaluated. Serum glucose, insulin, visfatin levels and peripheral CD3^+^, CD4^+/−^, CD8^+/−^ lymphocytes were assayed. All mice were alive and developed no metastasis, a greater number of obese mice developed palpable tumors than lean mice. The tumors from obese mice had a larger volume, greater intratumoral adipocyte mass, and exhibited a higher proliferation and reduced apoptosis rate compared to those of lean animals. Both serum insulin and visfatin concentrations correlated positively with tumor proliferation and negatively with tumor apoptosis. Obese mice had a significantly lower level of CD3^+^, CD3^+^CD4^+^ T lymphocytes, and a lower level of CD4^+^/CD8^+^ in peripheral blood compared to these lymphocyte levels in the lean mice. In conclusion, the altered adipocytokine milieu and insulin resistance observed in obesity may lead directly to alterations in the tumor microenvironment and cell immunity for avoiding cancer, thereby, promoting gastric cancer survival and growth.

## Introduction

Gastric cancer is a common lethal malignancy and treatment modalities for this advanced disorder remain limited. With the combination of high incidence and poor prognosis, gastric cancer holds the position of the fourth most common cancer and second most common cause of mortality in the world (1,2). Although the incidence of gastric cancer is declining in several Western countries, it is predicted that the number of gastric cancer cases globally will increase until 2050 (3). The virulence of gastric cancer is correlated with aggressive tumor biology and also indicates the current lack of effective systemic therapy. Dissecting the mechanisms of gastric cancer growth and progression is, therefore, critical to identify novel targets for desperately needed adjuvant therapies.

Obesity is a worldwide epidemic and more than one third of all adults are currently obese in the United States alone (4). White adipose tissue is an active endocrine organ, secreting a series of soluble mediators called adipocytokines that play an important role in regulating metabolism, inflammation and immunity. The altered adipocytokine milieu of obesity results in a generalized proinflammatory state and is a risk factor for developing systemic diseases, including diabetes, atherosclerosis and asthma (5). Notably, obesity has also been considered as a risk factor for developing numerous types of malignant tumors, including adenocarcinoma of the colon, prostate and breast (6). A meta-analysis demonstrated that the body mass index is closely correlated with the risk of gastric cancer, particularly cardia gastric cancer (7). Limited fundamental research exists regarding the mechanisms by which obesity affects gastric cancer biology and no *in vivo* animal models currently exist. Adopting this novel *in vivo* model, we sought to investigate the effect of obesity on gastric cancer growth and progression.

## Materials and methods

### Animals and cell culture

All experiments were carried out with approval from the Xi’an Jiaotong University Institutional Animal Care and Use Committee (Xi’an, China). Three- to five-week-old male C57BL/6j mice were obtained from Shanghai SLAC Laboratory Animal, Co., Ltd. (Shanghai, China), housed in standard conditions, divided into 2 groups and fed with a high-fat diet (35.5% fat, 36.3% carbohydrate, 20.0% protein) and a normal diet (5.4% fat, 51.0% carbohydrate, 22.9% protein) (8) for 12 weeks, respectively. The cancer cells used in animal studies, murine forestomach carcinoma cell line (MFC), were established in the Chinese Academy of Medical Sciences (9) and purchased from the Type Culture Collection of the Chinese Academy of Sciences (Shanghai, China). Cells were cultured in RPMI-1640 medium (Cellgro, Herndon, VA, USA) and supplemented with 10% fetal bovine serum (Valley Biomedical, Winchester, VA, USA), 1% penicillin/streptomycin and 1% glutamine (Cellgro).

### Experimental design

After 12 weeks, 12 mice consuming the normal-fat diet were referred to as ‘lean’, and 12/20 mice consuming the high-fat diet and chosen by the criterion that the body weight exceeded the mean plus 2-fold standard deviation (SD) of the lean mice were referred to as ‘obese’. All mice had insulin and glucose tolerance tests to confirm altered metabolism between the obese and lean mice. Then 2.0×10^6^ MFC cells were injected subcutaneously into the right flank. Mice were monitored daily, and the body weight of the mice and tumor sizes were measured every 3 days. After 2 weeks of tumor growth, both obese and lean mice were sacrificed after anesthesia and blood drawn from the retro-orbital venous plexus was preserved for analysis of metabolites and flow analysis of CD3^+^, CD4^+/−^, CD8^+/−^ T lymphocytes. Anti-mouse CD8a-APC, anti-mouse CD3e-FITC and anti-mouse CD4-PE monoclonal antibodies were purchased from eBioscience (San Diego, CA, USA). The tumors were carefully dissected and preserved in formalin for histological evaluation, as well as assay of proliferation and apoptosis. Laparotomy was performed to determine the existence of metastasis, which was confirmed by histology.

### Insulin and glucose tolerance tests

In order to determine the influence of obesity on glucose regulation and insulin sensitivity, we performed the insulin and glucose tolerance tests on obese and lean mice. The insulin tolerance test was performed at noon by intraperitoneal (i.p.) injection of 0.75 U/kg insulin, the i.p. glucose tolerance test was performed after overnight fasting by administering 20% glucose to mice (8). Blood glucose was measured using a Glucometer Elite (Bayer, Elkhart, IN, USA) at the indicated time points.

### Tumor characteristics

Cellular proliferation was assayed by DNA incorporation of 5-bromodeoxyuridine (5-BrdU). The mice were injected i.p. with 120 mg/kg 5-BrdU 1 h after sacrifice (10). A monoclonal 5-BrdU antibody and streptavidinbiotin staining system was used according to the manufacturer’s instructions (Santa Cruz Biotechnology, Inc., Santa Cruz, CA, USA), and the quantity of positively stained cells/10 high-power fields (original magnification, ×40) of formalin-fixed, paraffin-embedded tumor sections was analyzed.

Terminal deoxynucleotidyl transferase mediated deoxyuridine triphosphate nick-end labeling (TUNEL) assay (Roche Diagnostics, Brussels, Belgium) was used to identify and quantify apoptosis in formalin-fixed, paraffin-embedded tumor sections. Nuclear condensation, perinuclear clearing and cell shrinkage suggested TUNEL positivity and the number of cells/10 high-power fields was recorded. The number and extent of adipocytes and microvessel density were measured in 10 high-power fields of tumor sections that were stained with hematoxylin and eosin (H&E). All histological analyses were performed by three independent observers who were unaware of the tumor tissue source.

### Serum assays

Enzyme-linked immunosorbent assay (ELISA) was used to determine serum concentration of insulin (Crystal Chem, Inc., Downers Grove, IL, USA) and visfatin (Linco Research, Inc., St. Charles, MO, USA) following the manufacturer’s instructions. Serum glucose was determined by colorimetric assay.

### Statistical analysis

Values are expressed as the mean ± SD. Analysis of the data and plotting of the figures were performed with the aid of software (Origin version 7.5 and SPSS version 13.0). Analysis of variance and the Tukey’s test were applied where appropriate. P<0.05 was considered to indicate a statistically significant result.

## Results

### Metabolic changes in mice

**Figure 1:**
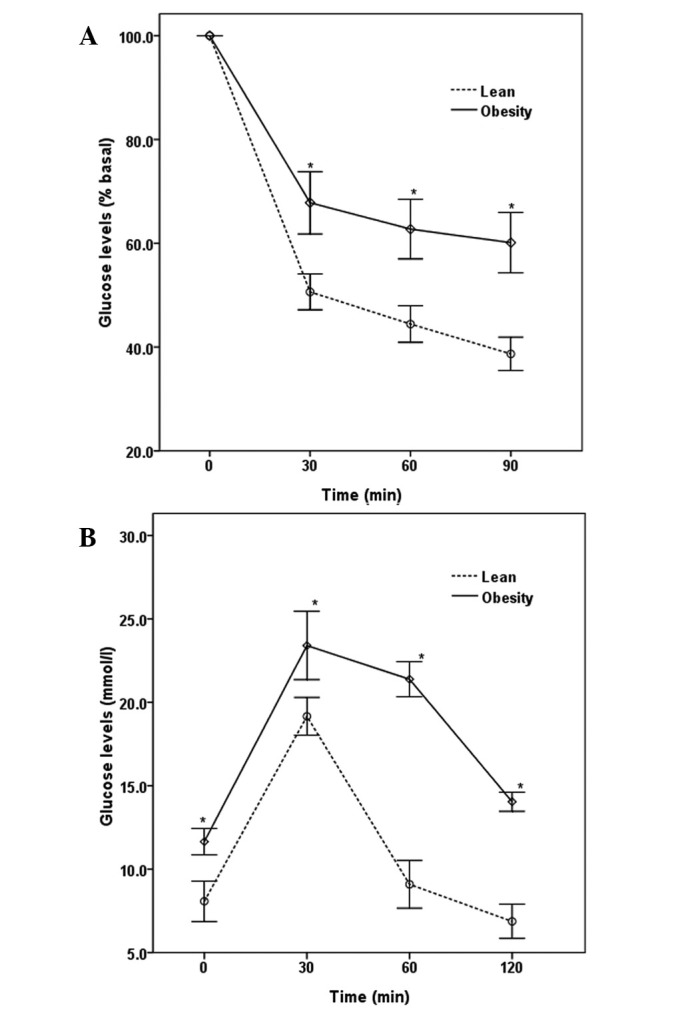
Diet-induced obesity led to several metabolic changes, and a state of (A) insulin resistance and (B) glucose tolerance existed in the obese mice. *P<0.05 vs. lean mice.

The mice were maintained on a normal or high-fat diet for 12 weeks. At the time of transplantation, the obese mice were significantly heavier than the lean animals (35.42±2.83 vs. 28.50±1.15 g; P<0.01). Several metabolic parameters were also altered in the obese mice, and those mice were insulin resistant and glucose intolerant (Fig. 1).

### Tumor growth, metastasis and mortality

**Figure 2:**
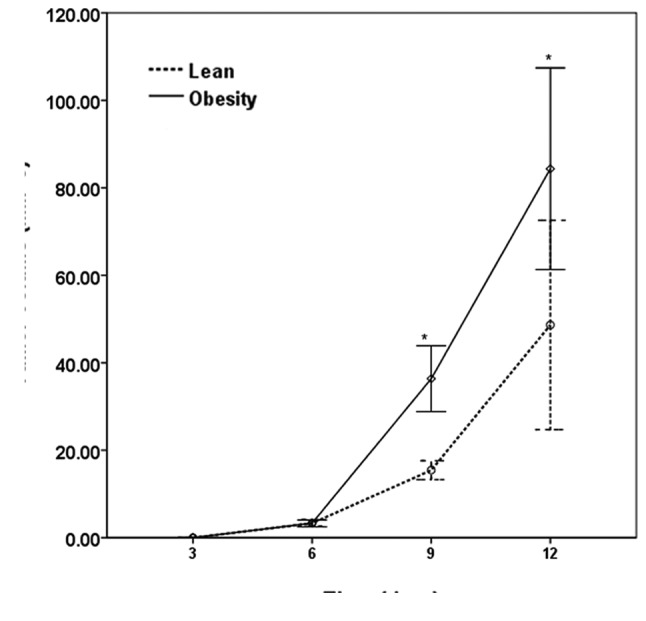
Tumor growth 2 weeks following flank injection. Tumor volume was measured in 3 dimensions. ^*^P<0.05 vs. lean mice.

**Figure 3:**
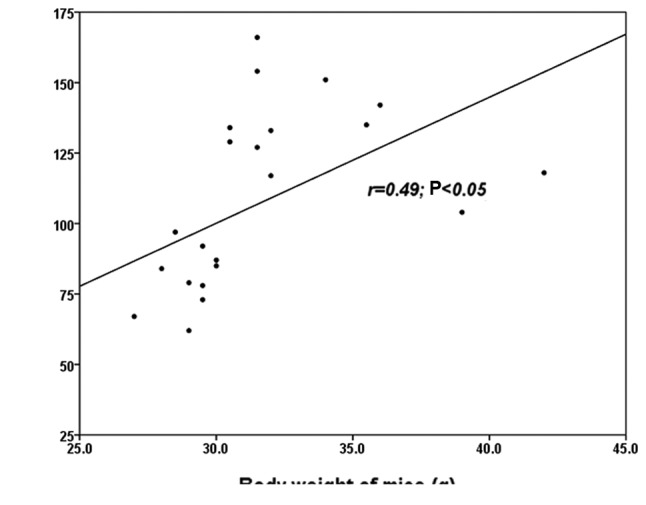
Correlation of body weight with weight of final excised tumor. r=0.49, P<0.05.

Injected animals were maintained on a normal- or high-fat diet for another 2 weeks. All mice were alive and metastases were not detected during the experimental time frame. The tumors became palpable 4 days after injection and tumor growth was observed in 10/12 (83.3%) of the lean mice and in 100% of the obese mice. Tumors grew larger and faster in the obese mice than those in the lean mice within 2 weeks (Fig. 2). Both obese and lean mice were then sacrificed after anesthesia and the tumors were preserved. The tumor weights were as follows: lean, 77.2±14.9 mg; obese, 134.2±17.3 mg (P<0.05 vs. lean). Tumor weight demonstrated a strong positive correlation with the body weight of the mice (r=0.49, P<0.05; Fig. 3).

### T lymphocytes in cell immunity

When tumors grew for 2 weeks, mice were sacrificed after anesthesia, and blood was collected to isolate lymphocytes for flow analysis of CD3^+^, CD4^+/−^, CD8^+/−^ T lymphocytes. Obese mice had a significantly lower level of CD3^+^, CD3^+^CD4^+^ T cells (P<0.05), and a lower level of CD4^+^/CD8^+^ in peripheral blood compared with these levels in the lean animals (P<0.05). No difference between obese and lean mice in regards to the levels of CD3^+^CD8^+^ T lymphocytes was observed (P>0.05; Table I).

### Proliferation and apoptosis

**Figure 4:**
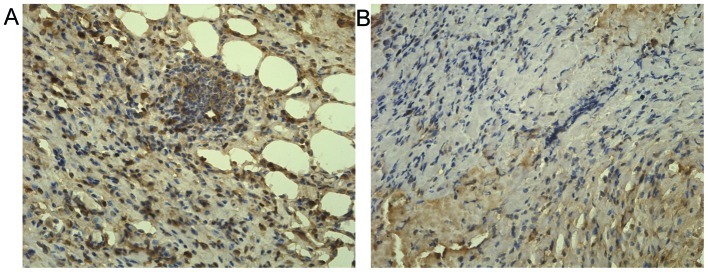
Representative micrographs (5-BrdU incorporation; original magnification, x40) indicating significantly increased cell proliferation in tumors from (A) obese mice compared to tumors from (B) lean animals (P<0.05).

**Figure 5:**
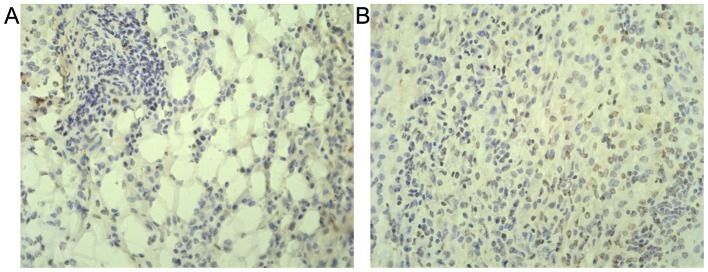
Representative micrographs (TUNEL; original magnification, x40) indicating significantly descreased cell apoptosis in tumors from (A) obese mice relative to tumors from (B) lean animals (P<0.05).

Tumor proliferation was measured by 5-BrdU uptake and apoptosis was measured by TUNEL assay. Cellular 5-BrdU uptake in tumors from lean and obese mice was as follows: 47.7±10.2 cells/hpf; 88.1±8.8 cells/hpf (P<0.01 vs. lean; Fig. 4). Cellular apoptosis in tumors from lean mice and obese mice were as follows: 54.7±5.7 cells/hpf; 34.7±4.6 cells/hpf (P<0.01 vs. lean; Fig. 5). These results demonstrated that increased tumor size in obese mice was not only correlated with apoptotic arrest, but also was a function of more rapid tumor proliferation.

### Metabolic parameters

Table II shows the results of serum glucose, insulin and visfatin concentrations, as well as the homeostatic model assessment (HOMA) score, which is a measure of insulin resistance. Obese mice were hyperglycemic, hyperinsulinemic and insulin resistant. Serum visfatin was increased in obese compared to that in the lean mice.

**Figure 6:**
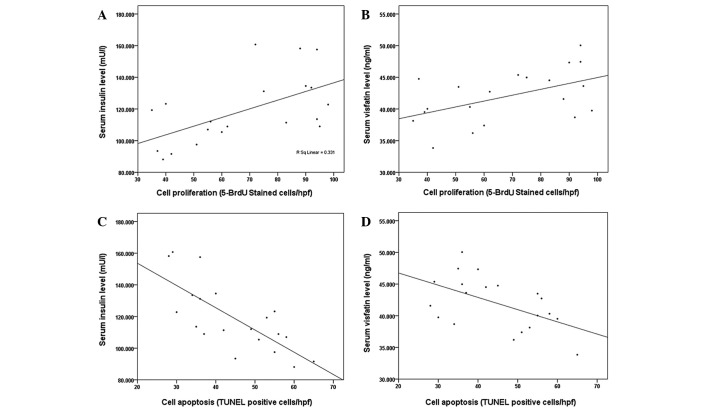
(A) Correlation between circulating insulin and tumor cell proliferation measured by 5-BrdU incorporation (r=0.58, P<0.05). (B) Correlation between serum visfatin and tumor cell proliferation measured by 5-BrdU incorporation (r=0.51, P<0.05). (C) Correlation between circulating insulin and tumor cell apoptosis measured by TUNEL (r=−0.74, P<0.05). (D) Correlation between serum visfatin and tumor cell apoptosis measured by TUNEL (r=−0.53, P<0.05).

Tumor proliferation correlated significantly with serum insulin (r=0.58, P=0.01; Fig. 6A) and visfatin concentrations (r=0.51, P=0.02; Fig. 6B), while it did not correlate with serum glucose concentration (r=0.20, P=0.1). Tumor apoptosis showed a strong negative correlation with circulating insulin (r=−0.74, P<0.01; Fig. 6C) and serum visfatin concentrations (r=−0.53, P=0.02; Fig. 6D), but not with glucose concentration (r=−0.16, P=0.25). The level of CD3^+^, CD3^+^CD4^+^ T cells did not correlate significantly with serum glucose, insulin or visfatin concentrations (P>0.05).

### Tumor microenvironment

**Figure 7:**
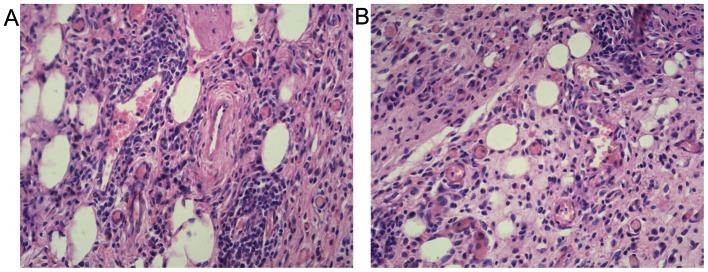
Representative micrographs (H&E; original magnification, ×40) indicating significantly increased adipose content within tumors of (A) obese mice compared to tumors of (B) lean mice (P<0.05). A great number of microvessels existed within the tumors; the microvessel density in tumors from obese mice was greater than that from lean animals without significance (A vs. B, P>0.05).

Microscopic analysis of tumors demonstrated an interesting observation. In addition to the fibrosis typically apparent in gastric cancers, a significant number of adipocytes were present within the tumors (Fig. 7). Although the average number of adipocytes exhibited no changes among tumors from obese and lean mice (17.2±4.2 cells/hpf vs. 15.4±2.7 cells/hpf, P>0.05), intratumoral adipocytes present in tumors from obese mice were significantly larger than those from lean animals (169.9±5.7 vs. 67.3±8.2 μm^2^, P<0.01). Another notable observation was that a great number of microvessels existed within the tumors. The microvessel density in the tumors from obese mice was greater than those from lean mice, but the difference was not significance (9.2±1.0 vs. 7.1±1.5/hpf, P>0.05).

## Discussion

This study is the first to use a completely novel *in vivo* animal model of gastric cancer growth in obesity. The fact that tumors grew larger and faster in obese mice relative to lean animals provides powerful evidence for the direct influence of obesity on gastric cancer growth. Rapid tumor growth was not only a function of decreased apoptosis, but was also correlated with increased cellular proliferation. Notably, tumor cell proliferation was positively correlated with serum visfatin and insulin concentrations, and tumor apoptosis showed a strong negative correlation with circulating visfatin and serum insulin concentrations. Tumor survival and growth in immunocompetent mice correlated with T lymphocyte levels involved in cell immunity; fewer CD3^+^, CD3^+^CD4^+^ T cells in peripheral blood from obese than lean mice led to tumors growing larger and faster in obese relative to lean mice.

A significant observation was the unexpected difference in tumor microenvironment. Tumors growing in obese mice had significantly greater adipocyte mass than tumors from lean animals. The effect of the tumor microenvironment on cancer growth and progression has become well understood, but the provocative concept that adipocytes active in metabolism may correlate with this milieu is completely novel and deserves further investigation. This model clearly constitutes a powerful instrument with which to further understand the mechanisms by which obesity affects gastric cancer growth.

Obesity always accompanies hypertriglyceridemia and hypercholesterolemia. Hypertriglyceridemia, but not hypercholesterolemia, was found to be an independent risk factor for lymph node metastasis in male patients of early gastric cancer, indicating that elevated serum TG levels may provide circumstances conducive to the development of lymph node metastasis in the early stage of gastric cancer, at least in male patients (11,12). The long-term survival of patients with gastric cancer is governed by the volume of intraperitoneal adipose tissue, and obese patients with stage 2 had a significantly lower mean survival rate than lean patients (13,14). Carcinogenesis in obese patients is determined by a range of important mechanisms and metabolites, including insulin, insulin resistance, inflammatory cytokines and visfatin (15,16), and a strong positive correlation was observed between serum insulin concentration and gastric cancer cell proliferation in this report. Despite these observations, the mechanisms by which obesity affects gastric cancer growth and progression remain completely unknown.

White adipose tissue was considered to be an inert tissue functioning solely as an energy store for a number of years, but has currently attracted increased attention for secreting adipocytokines as an endocrine tissue. Adipocytokines are small, hormonally active molecules that are structurally similar to cytokines and are produced mainly by adipocytes. These pleiotropic compounds exert a wide range of biological functions that include inflammation, immunity and other metabolic effects (5). As such, the altered adipocytokine milieu of obesity is a striking mechanistic link to potentiating tumor growth and progression. It is worthwhile to note the role played by visfatin in mediating insulin sensitivity, and this prominent adipocytokine may well prove to be an important mechanistic link in the network of factors affecting obesity-associated tumor growth.

Visfatin was originally identified as pre-B-cell colony-enhancing factor (PBEF), a putative cytokine isolated from peripheral blood lymphocytes, and described as a secreted growth factor for early B cell proliferation (17), and drew more attention after Fukuhara *et al* (18) reported it as an insulin-mimetic adipocytokine secreted by visceral fat. The circulating visfatin concentration increases with increasing obesity, and contributes to a general proinflammatory state in the periphery, and is gaining more attention and is a more widely studied adipocytokine in relation to cancer biology. To date, however, these studies have been limited to *in vitro* models. Generally, visfatin has been observed to potentiate tumor proliferation and metastasis in a variety of cancers including breast (19,20) and prostate (21). Common mechanistic pathways include activation of the extracellular signal regulated kinase (ERK1/2) pathway and phosphorylation of the signal transducer and activator of transcription 3 (STAT3) (22). In the current study, circulating visfatin concentration correlated with tumor progression in malignant astrocytomas (23), gastric cancer (24), colorectal cancer (25) and this report demonstrated the association with gastric cancer growth and visfatin, that played an important role in increasing gastric cancer cell proliferation and decreasing cell apoptosis.

One limitation of the murine model is that the MFC cell line originated from 615 mice, not from C57BL/6j. MFC was established in the Chinese Academy of Medical Sciences in the late 1980s by culturing small pieces of forestomach squamous cell carcinoma xenograft tumor from 615 mice. The 615 mice, with a partial gene background of C57BL/6j mice, were established in 1961 by the Blood Transfusion and Blood Institute, Chinese Academy of Medical Sciences (9). Therefore, C57BL/6j mice possessing a complete immune system may reject the MFC xenograft. However, we observed that the MFC xenograft injected subcutaneously in C57BL/6j mice grew larger in 2 weeks, then became smaller after a longer time. Also, this report investigated the changes in T lymphocytes in cell immunity correlating with MFC survival in C57BL/6j mice. Fewer CD3^+^, CD3^+^CD4^+^ T cells in peripheral blood from obese than lean mice led to tumor survival and increased growth in obese mice relative to lean mice. Another limitation of the murine model is the small volume of serum and tissue available for analysis. We measured the visfatin concentration which is relative to carcinogenesis and was recently identified as an adipocytokine. It is likely that some cytokines and adipocytokines are involved in regulating gastric cancer growth in obesity. Further studies are required to completely characterize the model.

In the present study, simple hematoxylin and eosin (H&E) histology indicated a completely striking and most provocative observation. Tumors that originated from the same cell type grew and exhibited a discrete physiological *in vivo* phenotype, and developed remarkably different microenvironments. Specifically, tumors from obese mice had significantly greater adipocyte mass than those tumors growing in lean mice. Adipose stromal cells promote tumor growth by secreting adipocytokines assisting in the formation of new blood vessels, a process necessary for the expansion of tumor mass, indicating that the tumor microenvironment in cancer may be modulated by white adipose tissue-derived trophic factors in a paracrine rather than in an endocrine manner, and stromal and vascular progenitor cells from white adipose tissue grafts were also associated with acceleration of cancer progression (26,27). The complicated interaction between extracellular matrix stromal cells and epithelial cells has attracted much recent attention, and several peices of evidence support the concept that aberrant signaling by extracellular matrix cells directly affects epithelial carcinogenesis and potentiates metastasis of malignant cells (28). As a result, we suggest that adipocytes active in metabolism play an important role in the process, and the potential to identify unique therapeutic targets is clear. In conclusion, these experiments show the first *in vivo* study of gastric cancer in the context of obesity. These data support the concept that insulin resistance and the altered adipocytokine milieu observed in obesity may lead directly to changes in the tumor microenvironment, thereby potentiating gastric cancer growth.

## Figures and Tables

**Table I:** T lymphocytes involved in cell immunity in the peripheral blood of the obese and lean mice.

Group	CD3^+^ (%)	CD3^+^CD4^+^ (%)	CD3^+^CD8^+^ (%)	CD4^+^/CD8^+^ (%)
Lean	37.44±6.10	19.49±3.71	15.99±2.96	1.23±0.18
Obese	32.23±2.86^a^	13.87±1.98^a^	17.47±1.95	0.80±0.13^a^

a P<0.05 vs. lean mice.

**Table II:** Serum metabolic parameters in mice.

	Group	
Parameters	Obese	Lean
Visfatin (ng/ml)	44.3±3.6^a^	39.6±3.4
Insulin (mU/l)	133.2±19.8^a^	104.7±11.8
Glucose (mmol/l)	11.9±1.6^a^	7.8±1.6
HOMA-IR	4.2±0.2^a^	3.8±0.2

a P<0.05 vs. lean. IR, insulin resistance; HOMA, homeostatic model assessment.
